# The endocannabinoidome–microbiota axis in obesity: a potential link to colorectal cancer development

**DOI:** 10.1007/s13105-026-01225-8

**Published:** 2026-08-21

**Authors:** Gabriela Ávila Alpino, Poliana Guiomar Brasiel, Mariana M. Almeida, Sheila C. P. Dutra Luquetti

**Affiliations:** 1 Departamento de Nutrição, Universidade Federal de Juiz de Fora, Juiz de Fora, Minas Gerais Brasil; 2 Departamento de Farmacologia, Universidade Federal de Juiz de Fora, Juiz de Fora, Minas Gerais Brasil

**Keywords:** Endocannabinoidome (eCBome), Endocannabinoids (eCBs), Intestinal microbiota, Short-chain fatty acids (SCFAs), Obesity, Dysbiosis, Colorectal cancer (CRC)

## Abstract

Colorectal cancer (CRC) is a multifactorial disease strongly influenced by genetic and environmental factors, as well as by immune and metabolic disorders such as obesity and gut dysbiosis. Recent evidence highlights the interplay between the endocannabinoidome (eCBome) and the gut microbiota as a central regulatory axis of intestinal homeostasis, metabolism, and immune responses. Although no studies have directly investigated this functional interaction in the CRC context, recent evidence suggests that dysfunction of the eCBome–microbiota axis, commonly observed in obesity, may influence colorectal tumorigenesis by impairing intestinal barrier integrity, increasing endotoxemia and immune dysregulation, altering microbial metabolite production, and activating NF-κB-mediated pro-tumorigenic signaling pathway. This is the first review to comprehensively integrate current evidence linking obesity-associated eCBome–microbiota dysfunction with CRC-related mechanisms and to propose potential pathways through which this interaction may influence CRC pathogenesis, providing a framework to guide future studies and support the development of novel preventive and therapeutic strategies for CRC.

## Introduction

Colorectal cancer (CRC) is the third most diagnosed cancer and the second leading cause of mortality worldwide [1]. Projections indicate that, by 2040, the global burden of CRC could reach 3.2 million new cases and 1.6 million deaths, representing a 63% increase in incidence and 72% increase in mortality compared with those in 2020 [2]. This rising incidence has been particularly pronounced in adults under 50 years.

Although family history is an important risk factor for early-onset CRC, approximately 80% of cases are linked to westernized dietary patterns and sedentary lifestyles, which promote metabolic dysfunction and favor the development of obesity [3]. Obesity is, in turn, considered a major risk factor for CRC development, as it induces chronic low-grade inflammation, adipokine imbalance, immune dysregulation, and gut dysbiosis that collectively contribute to genetic and molecular changes involved in colorectal carcinogenesis [1, 4].

Among these alterations, gut dysbiosis plays a central role in CRC pathophysiology. Characterized by alterations in microbial composition and function, often accompanied by reduced microbial diversity, dysbiosis disrupts intestinal homeostasis and promotes tumor development by activating oncogenic pathways, including WNT/β-catenin, while sustaining inflammation through NFκB, IL-17, and STAT3 signaling [5]. In addition, dysbiosis reduces the production of beneficial microbial metabolites, particularly short-chain fatty acids (SCFAs), impairing intestinal barrier integrity, amplifying inflammation, and promoting tumor progression [5].

Growing evidence indicates that obesity is also associated with dysregulation of the endocannabinoid signaling system [6–8], encompassing both the classical endocannabinoid system (ECS) and the expanded endocannabinoidome (eCBome). Importantly, recent evidence suggests a bidirectional interplay between the eCBome and the gut microbiota [9], raising the possibility that coordinated alterations in these systems represent a mechanistic link between obesity and colorectal carcinogenesis.

The classical ECS is a lipid signaling system widely distributed throughout the body and involved in the maintenance of physiological homeostasis, including the regulation of intestinal function [10]. Its principal mediators, anandamide (AEA) and 2-arachidonoylglycerol (2-AG), primarily activate cannabinoid receptor type 1 (CB1) and type 2 (CB2) to regulate intestinal permeability, barrier integrity, and inflammatory signaling pathway [9–11]. Expanding the classical ECS, the eCBome comprises a broader network of lipid mediators and their molecular targets that extends endocannabinoid signaling. Beyond its role in intestinal and metabolic homeostasis, the eCBome is increasingly recognized as a key modulator of host–microbiota communication [9, 12–15].

Given the central roles of the eCBome and the gut microbiota in maintaining intestinal homeostasis, together with emerging evidence supporting bidirectional communication between these systems, obesity-induced dysregulation of both may cooperatively disrupt intestinal function, thereby representing a previously underexplored mechanistic link between obesity and colorectal carcinogenesis. However, no experimental studies have directly investigated the eCBome-microbiota interaction in the context of CRC. In addition, the molecular mechanisms involved in the communication between these systems remain poorly understood.

Therefore, the aim of this review is to explore the mechanisms underlying this interaction, with an emphasis on the inflammatory processes associated with obesity given its relevance as a risk factor for CRC. We also aim to provide new insights that support future investigations into the molecular pathways related to the eCBome-microbiota-CRC triad and contribute to the development of innovative therapeutic and preventive strategies of CRC. The scope of this review is the endocannabinoid signaling, whereas phytocannabinoids and synthetic cannabinoids are not included.

## The role of eCBome in intestinal homeostasis

**Fig. 1:**
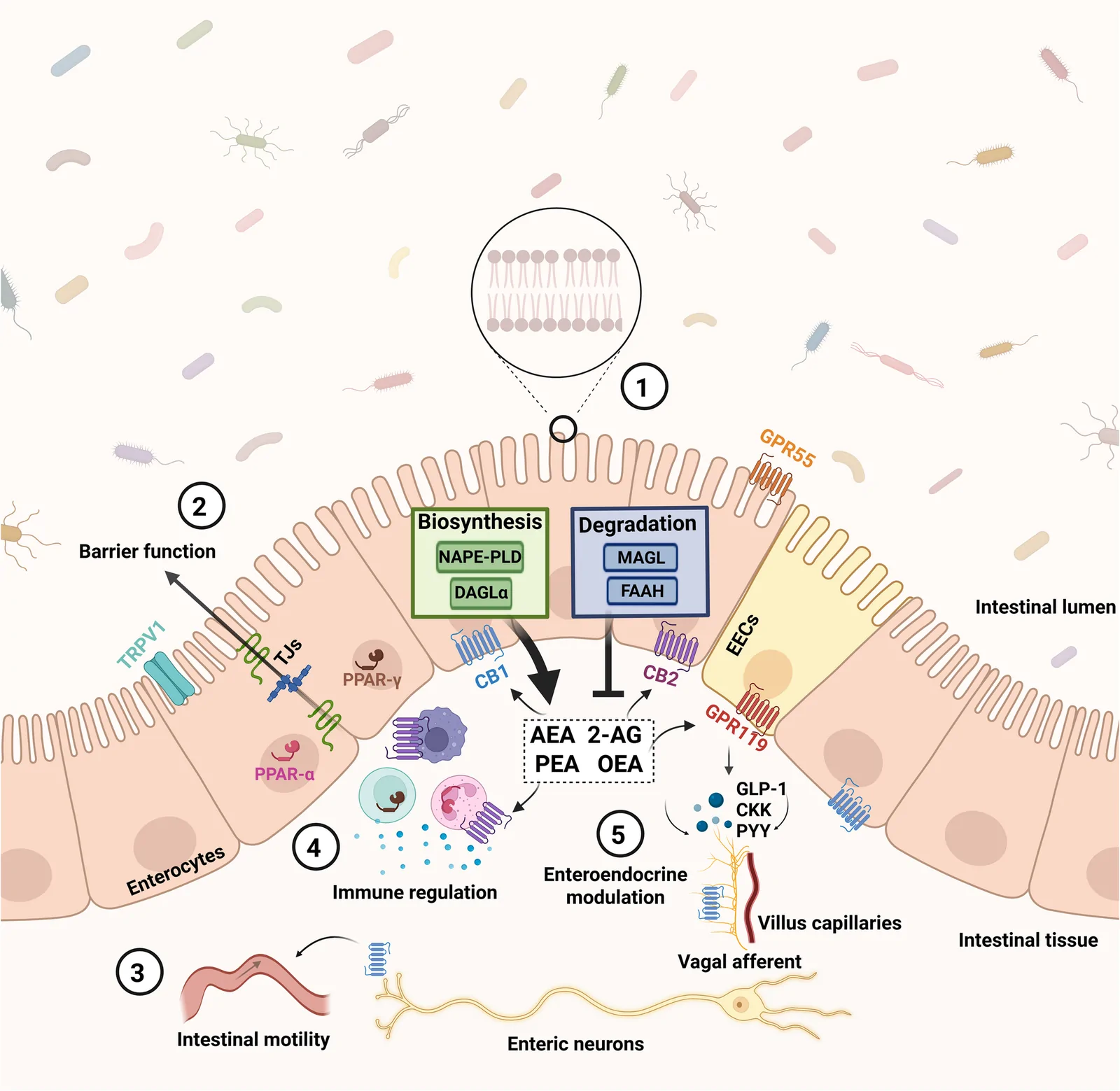
Role of eCBome in intestinal homeostasis. Classical endocannabinoids (AEA and 2-AG) and eCBome-related lipid mediators (PEA and OEA) are generated from membrane phospholipid precursors **through biosynthetic pathways involving NAPE-PLD and DAGLα and are primarily degraded by FAAH and MAGL** (1). Upon binding to their respective receptors, including CB1, CB2, TRPV1, GPR55, PPAR-α, and PPAR-γ, these mediators contribute to the regulation of intestinal barrier integrity through modulation of TJs (2), intestinal motility via CB1 signaling in enteric neurons (3), local immune responses through CB2 and PPAR signaling in immune cells (4), and enteroendocrine functions mediated by receptors expressed in EECs (5). **eCBome**, endocannabinoidome; **AEA**, anandamide; **2-AG**, 2-arachidonoylglycerol; **PEA**, palmitoylethanolamide; **OEA**, oleoylethanolamide; **NAPE-PLD**, N-acyl phosphatidylethanolamine phospholipase D; **DAGLα**, diacylglycerol lipase α; **FAAH**, fatty acid amide hydrolase; **MAGL**, monoacylglycerol lipase; **CB1**, cannabinoid receptor 1; **CB2**, cannabinoid receptor 2; **TRPV1**, transient receptor potential vanilloid 1; **GPR55**, G protein-coupled receptor 55; **GPR119**, G protein-coupled receptor 119; **PPAR**, peroxisome proliferator-activated receptor; **TJs**, tight junctions; **EECs**, enteroendocrine cells; **GLP-1**, glucagon-like peptide-1; **CCK**, cholecystokinin; **PYY**, peptide YY. Created in https://BioRender.com

The eCBome plays a central role in maintaining intestinal homeostasis by regulating multiple physiological processes, including intestinal motility, epithelial barrier integrity, mucosal immune responses, and enteroendocrine signaling (Fig. 1), as discussed in the following subsections.

### eCBome regulation of intestinal motility

In the gastrointestinal (GI) tract, CB1 is mainly expressed in enteric neurons, afferent nerves, and epithelial cells (e.g., enteroendocrine and absorptive types) [11, 16]. The activation of CB1 promotes mesenteric vasodilation, inhibits fluid and acid secretion and stimulates intestinal motility [17]. CB2 receptors are absent on most healthy intestinal epithelial cells but are found mainly on subepithelial immune cells such as intestinal macrophages [18]. The expression patterns of both receptors in the GI tract vary according to the intestinal pathophysiological state. Under physiological conditions, intestinal ECS activity is predominantly mediated by CB1, whereas inflammation also activates CB2, triggering counterregulatory responses to mitigate inflammation [18, 19].

CB1 plays a key role in inhibiting GI motility and maintaining transit homeostasis. Peripheral activation of CB1 with selective agonists suppresses intestinal motility under physiological conditions and counteracts stress-induced acceleration of transit in animal models (27). Conversely, inhibition of CB1, particularly in vagal afferent neurons, increases GI motility and is often associated with diarrhea in rodents and humans [17, 20, 21]. Additionally, inhibition of FAAH also reduces motility in mice, suggesting that enhancing endocannabinoids (eCBs) tone may be beneficial in motility disorders [22]. Notably, AEA and 2-AG have been shown to inhibit myogenic contractions of human intestinal muscle through CB-independent mechanisms [23]. While CB1 primarily regulates motility under normal conditions, CB2 plays a more relevant role in pathological states, and its activation restores intestinal function by normalizing inflammation-induced hypercontractility [24].

### eCBome control of epithelial barrier integrity

The eCBome regulates the integrity of the intestinal epithelial barrier, influencing its permeability and, consequently, nutrient absorption and the interaction between the luminal environment and the mucosal immune system. CB1-mediated overactivation is associated with increased permeability, which is regulated by tight junction (TJ) proteins, such as claudins, occludins and zonulins (ZO) [25]. Moreover, SCFAs, especially butyrate, strengthen barrier integrity by regulating TJ proteins and mucus production. TJ disruption increases permeability, allowing lipopolysaccharide (LPS) translocation and activation of TLR4/NF-κB signaling, thereby sustaining chronic inflammation and promoting a pro-tumorigenic intestinal [26, 27].

In *ob/ob* mice, treatment with a CB1 antagonist led to a reduction in plasma LPS levels, an effect associated with altered distribution of ZO-1 and occludin and decreased intestinal permeability. The authors also reported that co-treatment with a CB1 agonist (HU210) and LPS impaired barrier function, as evidenced by reduced transepithelial electrical resistance (TEER) and the expression of TJ proteins in Caco-2 cells, a colon cancer-derived cell [26]. Similarly, apical application of AEA and 2-AG dose-dependently increased intestinal permeability in Caco-2 cells under both basal and inflammatory conditions via a CB1-dependent mechanism [28, 29].

In contrast, in vivo studies suggest that 2-AG acts as a ‘’gatekeeper’’ by reinforcing intestinal barrier function in models of obesity [11], colitis [30], and stress [31]. The endocannabinoid-related mediators 2-oleoylglycerol (2-OG), palmitoylethanolamide (PEA), and oleoylethanolamide (OEA), also act as gatekeepers [32–34], whereas AEA primarily acts as a “gate opener,” increasing permeability, particularly under inflammatory conditions [33, 34]. These findings suggest that CB1-mediated signaling varies depending on the ligand (e.g., AEA e 2-AG) and may also be influenced by the pathophysiological state of the intestine, the level of eCBs tone, and CB1 polarization between the apical and basolateral domains of epithelial cells, thereby activating distinct signaling pathways and producing differential effects on barrier function [35]. Moreover, the eCBome appears to regulate the intestinal barrier through CB1-independent mechanisms, such as modulation of the local immune system, which requires further investigation.

### eCBome modulation of mucosal immunity

The eCBome exerts immunomodulatory effects by interacting with receptors and ion channels expressed on intestinal immune cells, thereby regulating mucosal immune responses. These effects are primarily mediated through the activation of CB2, peroxisome proliferator-activated receptor alpha (PPARα), G protein-coupled receptor 55 (GPR55), and the transient receptor potential vanilloid 1 (TRPV1) channel [10]. For example, PEA binds to PPARα, which suppresses NF-κB activity, reducing inflammatory mediators like TNF-α, IL-1β, iNOS, and COX-2, and increasing the activity of antioxidant enzymes, such as catalase and superoxide dismutase (SOD), thus modulating intestinal inflammation [36, 37]. Consistently, PEA reduced inflammatory mediators in biopsy samples from patients with colitis and immune cell infiltration in colitis-induced rats [38]. The activation of CB2 controls the number of tolerogenic macrophages and promotes the expansion of regulatory T cells (Treg), allowing the immune system to differentiate pathogens from harmless antigens. This process is crucial for avoiding unnecessary inflammatory responses against the gut microbiota and maintaining immune homeostasis [39]. In addition, CB2 may also modulate immune responses by attenuating enteric glial activation and reducing TLR4 expression, although the mechanisms underlying these effects remain to be fully elucidated [11].

### eCBome regulation of enteroendocrine signaling

The eCBome regulates the production and secretion of intestinal hormones, such as glucagon-like peptide-1 (GLP-1), cholecystokinin (CCK), and peptide YY (PYY), through CB1 signaling, thereby influencing gastric emptying, digestion, nutrient absorption, energy metabolism, and anorexigenic responses [40]. Overactivation of intestinal CB1 inhibits the release of CKK and GLP-1 by enteroendocrine cells (EECs), which are related, respectively, to obesity-associated hyperphagia and the suppression of insulin secretion in the fed state [41]. Consistently, both AEA and 2-AG have been shown to increase food intake via CB1 activation, an effect that can be reversed by CB1 antagonists, supporting their role in the modulation of feeding behavior [39, 40]. 2-AG appears to be particularly sensitive to nutritional status, with increased levels during fasting and decreased levels following the intake of palatable foods, suggesting a role in the initiation of food intake [42], whereas AEA helps to prolong feeding beyond physiological need. In contrast, the activation of GPR119 by OEA stimulates GLP-1 secretion in vitro and in vivo [43, 44], while reducing food intake and promoting satiety for up to 24 h in free-feeding rats after oral administration [45].

## The role of gut microbiota and eCBome in intestinal disorders: links to CRC risk

The gut microbiota controls inflammation through multiple mechanisms, including modulation of innate immunity via the production of immunoglobulin A (IgA), α-defensins, lysozymes, and the mucus barrier, as well as regulation of adaptive immunity through Treg maturation and modulation of Th1 and Th17 cells [46]. Together, these processes maintain immune tolerance, allowing the immune system to distinguish commensal from pathogenic microorganisms.

Disrupted of host-microbiota interactions, combined with genetic susceptibility, contributes to the development of inflammatory bowel diseases (IBD), including ulcerative colitis (UC) and Crohn’s disease (CD), both characterized by chronic intestinal inflammation [47]. In turn, chronic inflammation, exacerbated by increased intestinal permeability and dysbiosis, drives the production of TNF, IL-17, IL-23, IFN-γ, and IL-6, reactive metabolites, and growth factors that collectively promote epithelial damage and create a microenvironment conducive to adenoma formation [18].

Individuals with IBD are approximately twice as likely to develop CRC compared to the general population [48]. In UC, CRC risk increases with disease activity, duration, severity, and extent of colonic involvement, with pancolitis conferring a 5- to 15-fold higher risk [49]. In CD, although the association with CRC is less clear, patients with colonic involvement and disease duration over 10 years have an annual CRC incidence of 0.5–1% [50]. Although irritable bowel syndrome (IBS) is not classified as an IBD, it has also been associated with dysbiosis, which may influence pathways relevant to CRC susceptibility [51]. A meta-analysis reported that patients with IBS have an approximately 2.8-fold higher risk of developing CRC than individuals without IBS [52], although the biological basis of this association remains uncertain.

Given the characteristics of intestinal disorders, particularly the inflammatory environment of IBD, investigating the eCBome–microbiota axis in theses contexts may provide biologically plausible insights into how this system could regulate mucosal inflammation, barrier integrity, and ultimately CRC risk. Evidence from experimental models of colitis and IBS may also offer initial mechanistic insights into these interactions.

Esposito et al. [53] investigated the effects of a genetically modified *Lactobacillus paracasei* strain carrying the gene encoding NAPE-PLD in a mouse model of colitis. The probiotic was capable of producing PEA directly in the GI tract, leading to significant improvements in colonic injury, reduced expression of inflammatory mediators, and modulation of TJ proteins via PPARα activation [53]. PEA does not directly bind CBRs but modulates mediators acting on these receptors, offering an additional mechanism for regulating intestinal inflammation and tissue repair. Although this study did not assess changes in gut microbiota composition, it is plausible that PEA’s effects were at least partly mediated by shifts in the microbial profile, as shown by Pirozzi et al. [54].

Notably, PEA has been shown to exert beneficial effects in intestinal disorders, primarily mediated through PPARα [36, 38, 53, 54]. Activation of this receptor regulates signaling pathways that promote gut microbiota homeostasis and mucosal immunity by modulating Th1 and Th17 responses, reducing inflammatory mediators such as IFN-γ, IL-1β, IL-6, and TNF-α, and enhancing the expression of IL-22 and antimicrobial peptides (AMPs), including α-defensins [55–57].

It is reasonable that the beneficial effects of PEA in intestinal disorders may be partially mediated by the gut microbiota, given the well-established role of PPARα signaling in regulating mucosal immunity and microbiota homeostasis. However, this hypothesize remains to be experimentally validated. Additionally, although endogenous PEA rises in response to inflammation in the GI tract, it is insufficient to restore intestinal homeostasis [55], highlighting the potential of exogenous supplementation.

Other N-acylethanolamines (NAEs), such as AEA, OEA, and linoleoyl ethanolamide (LEA), are typically elevated in the feces of patients with IBD. LEA and OEA have been shown to promote the growth of IBD-associated bacteria, including *Escherichia coli*, *Ruminococcus gnavus*, and *Blautia producta*, whereas LEA and AEA inhibit the growth of IBD-depleted species, such as *Bacteroides fragilis* in vitro [58]. Transcriptomic profiling of *B. fragilis* revealed increased expression of efflux proteins and reduced expression of genes involved in NAE transport and catabolism, suggesting adaptive mechanisms that limit intracellular NAE accumulation. Furthermore, ex vivo analysis showed that specific NAEs combination could shift a healthy microbiota toward an IBD-like profile [58]. Collectively, these findings suggest that elevated NAEs promote pro-inflammatory microbial changes, contributing to IBD pathogenesis. Thus, targeting NAE metabolism or receptor interactions may represent a promising strategy to modulate dysbiosis, reduce inflammation in IBD, and prevent long-term complications such as CRC.

The eCBome and gut microbiota regulate visceral sensory and motor functions that are frequently altered in IBS and IBD. As previously discussed, cannabinoid receptors modulate GI motility and visceral nociception [17, 59, 60], whereas microbiota-derived SCFAs regulate PYY and GLP-1 secretion through free fatty acid receptors (FFARs) FFAR2/GPR43 and FFAR3/GPR41, influencing intestinal transit [61]. These regulatory mechanisms contribute to epithelial homeostasis, and their disruption may promote chronic inflammation, favoring the formation of a pro-tumorigenic intestinal microenvironment.

SCFAs and the eCBome also exert complementary effects on metabolic and immune pathways involved in intestinal physiology. In the study by Vijay et al. [62], aerobic exercise-induced increases in SCFA-producing were shown to elevate levels of AEA, OEA, and PEA. Notably, AEA mediated approximately 30% of the butyrate-induced reduction in TNF-α levels, while 2-AG and OEA positively correlated with the anti-inflammatory cytokine IL-10. Furthermore, AEA and OEA were positively associated with both microbial alpha diversity and SCFA-producing taxa such as *Bifidobacterium* and *Faecalibacterium*, and showed positive correlation with expression of FFAR2 [62]. These findings indicate a functional cross-talk through which SCFAs modulate immune responses, partly via activation of eCBome. Despite evidence of interaction between SCFAs and the eCBome, the mechanisms underlying this interplay remain unclear. Nonetheless, these data suggest that restoring SCFA-eCBome signaling may represent a promising strategy to mitigate chronic intestinal inflammation and limit the establishment of a pro-tumorigenic intestinal microenvironment.

Notably, SCFAs stimulate serotonin (5-HT) release from enterochromaffin cells, increasing intestinal motility and modulating visceral sensitivity via activation of enteric neurons and vagal afferents [63]. Moreover, PEA has been shown to increase *Turicibacter sanguinis*, a species associated with higher colonic 5-HT levels, possibly through SCFA-mediated mechanisms [54]. These findings further support functional interactions between the eCBome and gut microbiota in IBS, a disorder characterized by altered colonic 5-HT signaling [64]. Importantly, intestinal 5-HT has been shown to suppress tumor initiation and inflammation during early stages of colitis-associated colorectal cancer in mice [65], suggesting that the SCFA–eCBome–5-HT axis may also contribute to CRC prevention. In addition, *Lactobacillus* modulates intestinal sensory and motor function via CB2 upregulation, reducing visceral hypersensitivity and enhancing colonic motility in rodents [59, 66]. Collectively, these findings support the potential role of the eCBome–microbiota axis not only in IBS-related dysfunction but also in processes involved in inflammation-associated colorectal carcinogenesis.

## Interplay between the eCBome and gut microbiota in metabolic disorders

The first evidence of interaction between the gut microbiota and the ECS was reported by Rousseaux et al. [59], who demonstrated that *Lactobacillus acidophilus* modulates visceral pain and intestinal homeostasis through CB2 signaling in rodents. Since then, several studies in animals and humans have confirmed this interaction, mainly in the context of obesity and associated metabolic disorders [32, 67–70], conditions in which these systems play central roles in regulating energy metabolism, inflammatory responses, and intestinal barrier function.

For example, the probiotic strain *Akkermansia muciniphila* was associated with increased levels of 2-AG and 2-oleoylglycerol (2-OG) in the ileum of high-fat diet (HFD)-induced obese mice [32]. These acylglycerols support intestinal barrier integrity, and lower levels of the 2-AG-degrading enzyme MAGL are associated with improved barrier function and reduced LPS-induced inflammation [25]. Moreover, *A. muciniphila* reversed the HFD-induced reduction of MAGL levels in the intestinal mucus layer and improved metabolic dysfunction, suggesting that this probiotic may help restore intestinal barrier function through modulation of the eCBome in obesity [32]. Corroborating these findings, *Lactobacillus johnsonii* improved intestinal barrier function in piglets by increasing occludin, ZO-1, and DAGL-β expression, enhancing motility, and reducing postweaning diarrhea [70].

Blocking CB1 has been shown to improve intestinal barrier function in diet-induced obese mice, as evidenced by a thicker mucus layer, reduced inflammation, and lower blood glucose levels and insulin resistance (IR). These effects were associated with an increased abundance of *A. muciniphila* and reduced abundances of *Lachnospiraceae* and *Erysipelotrichaceae* [71]. Conversely, germ-free mice exhibit increased colonic CB1 expression, along with alterations in enzymes involved in eCB synthesis and degradation, as well as overall eCB levels along the GI tract, compared to conventionally raised mice. Notably, these changes are partially reversed by fecal microbiota transplantation (FMT), with colonic *CNR1* expression returning to baseline after FMT [67].

These findings highlight a cooperative interaction between the gut microbiota and the eCBome in obesity. Obesity-associated dysregulation of both systems may impair intestinal barrier integrity and promote intestinal dysfunction, whereas interventions targeting either system can influence the other, supporting the existence of a bidirectional network involved in maintaining intestinal homeostasis.

Additionally, genetic disruption of key eCBome enzymes has been shown to alter gut microbiota composition and metabolic outcomes in obesity. Mice with adipose tissue-specific deletion of of the NAE biosynthetic enzyme NAPE-PLD exhibited reduced NAE levels, decreased abundance of *Lactobacillus* and *Allobaculum*, and increased weight gain, inflammation, and metabolic dysfunction [72]. Similarly, intestinal epithelial NAPE-PLD knockout mice led to reduced NAE levels and changes in the gut microbiota, together with increased food intake, decreased energy expenditure, and aggravated obesity and hepatic steatosis in HFD-fed mice [73]. Interestingly, mice lacking the eCB-degrading enzyme MAGL were protected against HFD-induced obesity and liver steatosis, an effect associated with increased abundance of *Hydrogenoanaerobacterium*,* Roseburia*, and *Ruminococcus* [74], further indicating that eCBome enzymatic tone regulates gut–microbiota–metabolic interactions.

In an HFD-induced nonalcoholic steatohepatitis (NASH) model, resveratrol (RSV) modulated gut microbiota and ECS, reducing LPS-producing *Desulfovibrio* while increasing beneficial taxa such as *A. muciniphila*, *Ruminococcaceae*, and *Lachnospiraceae* [75]. RSV also reversed HFD-induced ECS alterations by decreasing colonic *CNR1* and increasing *CNR2* expression. Additionally, RSV reduced DAGLα and FAAH expression and increased MAGL and NAPE-PLD levels, suggesting higher 2-AG and lower AEA levels. These changes were linked to improved intestinal barrier integrity and reduced metabolic endotoxemia. Notably, microbiota depletion or pharmacological blockade of ECS signaling abolished RSV’s protective effects, highlighting the interconnected role of the gut microbiota and ECS in mediating these benefits [75].

Administration of PEA to HFD-fed mice resulted in a decreased Firmicutes–Bacteroidetes ratio, reversing obesity-associated dysbiosis. This shift included an increase in SCFA-producing taxa such as *Bifidobacterium*, *Romboutsia*, and *Turicibacter*. These microbial changes were associated with modulation of tryptophan metabolism and a marked reduction in colonic inflammation, as evidenced by decreased levels of TLR4, COX-2, iNOS, TNF-α, and IL-1β, contributing to improved intestinal homeostasis [54].

Maternal obesity induced by a high-fat-high-sugar (HFHS) diet reduced fecal levels of PEA and OEA in offspring at weaning and was associated with obesity and impaired glycemic homeostasis. This was accompanied by changes in other fecal metabolites, suggesting altered microbial function, although microbiota composition was not directly assessed [34]. Together, these findings indicate that maternal HFHS exposure programs long-term alterations in the offspring’s intestinal eCBome, potentially contributing to early-life metabolic dysfunction through eCBome–microbiota interactions, which warrants further investigation.

Similary, Lacroix et al. [76] showed that HFHS diets induce rapid and concurrent changes in both the eCBome and gut microbiota in adult mice. Within just three days, HFHS reduced microbial diversity, increasing Firmicutes and decreasing Bacteroidetes, along with expansions of *Lactococcus* and *Romboutsia* and reductions in *Alistipes* and *Bacteroides*. In parallel, lipid mediator profiles shifted: ileal AEA levels increased by day 10, whereas PEA and OEA declined early. In plasma, AEA and 2-AG increased by days10 and 21, while OEA, 2-OG, and omega-3-derived mediators decreased [76]. These coordinated changes coincided with microbiota shifts, suggesting early crosstalk between the eCBome and gut microbiota preceding overt obesity. Overall, these findings suggest that obesogenic diets may acutely disrupt this axis before the development of metabolic disease.

In humans, short-term dietary modulation has been shown to rapidly influence the eCBome–microbiota axis. A 2-day Mediterranean diet (MD), rich in omega-3 polyunsaturated fatty acids and oleic acid, increased circulating NAEs and 2-MAGs in overweight and obese individuals, independently of adiposity. Notably, changes in bacterial families such as *Veillonellaceae*, *Peptostreptococcaceae*, and *Akkermansiaceae* were associated with omega-3–derived NAEs and 2-MAGs, independent of diet and adiposity, supporting a role for the gut microbiota in regulating eCBome signaling beyond these factors [68].

Similarly, an 8-week MD intervention reduced plasma AEA and increased OEA/PEA and OEA/AEA ratios, along with increased faecal *Akkermansia muciniphila* in overweight and obese individuals, independent of body weight changes. These alterations were associated with improved insulin sensitivity and reduced systemic inflammation [77], supporting the translational relevance of MD-induced modulation of *A. muciniphila* and eCBome signaling. Collectively, these human studies highlight the translational relevance of diet-induced modulation of the eCBome–microbiota axis, linking short- and medium-term dietary interventions to metabolic, inflammatory, and intestinal health outcomes.

Additionally, plasma AEA and related NAEs were positively correlated with *Faecalibacterium* and *Akkermansia* and negatively associated with *Bilophila*, whereas 2-AG and other acylglycerols showed positive correlations with *Parasutterella* and *Odoribacter* and negative associations with *Prevotella* in healthy young adults [75]. These observational associations were accompanied by correlations with markers of intestinal barrier integrity and inflammatory regulation, suggesting a potential link between the eCBome–microbiota axis and intestinal homeostasis in healthy adults.

The bidirectional interplay between the eCBome and the gut microbiota remains incompletely understood, particularly at the mechanistic level. Current evidence is largely context-dependent, with a predominant focus on obesity, which limits broader physiological and pathological interpretation. This highlights the relevance of investigating how these systems interact across different health and disease states.

## From obesity to CRC: the eCBome–microbiota axis as a potential link

Obesity, resulting from caloric imbalance, is a key risk factor for CRC [1]. Evidence from both animal and human studies indicates that obesity is associated with alterations in the eCBome and the gut microbiota [54, 68, 76, 78], suggesting that disruption of the eCBome–microbiota axis may constitute a pathophysiological link between obesity and CRC.

A central feature linked to this metabolic state is ECS hyperactivation, as evidenced by increased CB1 expression, elevated levels of AEA, and reduced concentrations of PEA and OEA [76, 78]. HFDs increase the availability of fatty acids, which serve as precursors for eCBs synthesis, leading to ECS hyperactivation primarily through the CB1 [79, 80]. Overstimulation of ECS triggers metabolic disturbances typical of obesity, such as hyperphagia, enhanced lipogenesis, reduced lipolysis and energy expenditure, resistance to insulin and leptin, along with intestinal dysbiosis, resulting in systemic metabolic dysfunction and chronic inflammation (85).

Dysbiosis may favor the abundance of protumorogenic pathogens associated with CRC, such as *Fusobacterium nucleatum*, enterotoxigenic *Bacteroides fragilis* and *Escherichia coli pks+* [5]. For example, *Escherichia coli pks+* produce genotoxins, such as *cytolethal distending toxin* (CDT), which cause DNA damage and induce genetic changes capable of activating oncogenes and/or inactivating tumor suppressor genes. In addition, *F. nucleatum* expresses the adhesin FadA, which activates oncogenic pathways such as the WNT/β-catenin pathway [5].

Pathogen-associated molecular patterns (PAMPs) constitutes a critical link between obesity-induced dysbiosis and colorectal tumorigenesis. PAMPs such as LPS and lipoteichoic acids activateS the TLR4/R2 signaling pathways, respectively. This activation, mediated by the MYD88 adapter, leads to NFκB increases of IL-6, IL-1β, and TNF-α [81–83]. The inflammatory cascade is further amplified by STAT3 activation, which promotes IL-17 and IL-23 production, sustaining chronic inflammation via Th17 cells and neutrophil-derived ROS, thereby enhancing cell proliferation, angiogenesis, and resistance to apoptosis [18].

Interestingly, MyD88 deletion has been associated with reduced AEA and increased levels of 2-AG, resulting in anti-inflammatory effects by enhancing intestinal Treg cells and reducing IL-6 and resistin in HFD–induced obese mice [84].

Additionally, harmful bacterial metabolites, such as hydrogen sulfide (H₂S) and the secondary bile acids deoxycholic acid (DCA) and lithocholic acid (LCA), promote tumorigenesis by disrupting the colonic epithelial barrier, inducing NF-κB activation, and increasing oxidative stress, ultimately leading to genomic instability [18]. These deleterious effects are further exacerbated by the loss of SCFA-producing bacteria, which normally support epithelial barrier integrity [85]. Consequently, increased intestinal permeability enhances bacterial translocation, amplifies intestinal and systemic inflammation, and ultimately establishes a pro-tumorigenic environment that supports colorectal cancer development and progression.

As previously described, certain probiotics such as *A. muciniphila* and *L. johnsonii* contribute to the maintenance of intestinal barrier integrity by increasing gatekeeper mediators [32, 70]. Notably, the abundance of *A. muciniphila* and *L. johnsonii* is often reduced in obesity and CRC, and their supplementation has been shown to inhibit colorectal tumorigenesis in preclinical models [85–88]. However, the extent to which these effects involve eCBome modulation is largely unknown, representing a key limitation in the current evidence. We speculate that these probiotics may exert antitumor actions, at least in part, through their ability to modulate the eCBome–intestinal microbiome axis, potentially strengthening intestinal barrier function and highlighting a mechanistic link that warrants further investigation.

Reciprocally, eCBome modulation in obesity may promote a protective intestinal microbial profile against associated diseases such as CRC. For example, OEA supplementation (250 mg/day) increased the abundance of *A. muciniphila* and reduced energy intake in obese individuals compared with individuals in the control group [89]. Similarly, pharmacological blockade of CB1 has been shown to modulate the intestinal microbiota in a diet-induced obesity model, leading to an increased abundance of *A. muciniphila*, reduced metabolic inflammation and improved intestinal barrier function [71]. These findings reinforce the potential of eCBome-targeted interventions to beneficially reshape gut microbiota communities and prevent obesity-associated diseases such as CRC.

In summary, obesity induces alterations in both the eCBome and the gut microbiota, which are interconnected through mechanisms that remain incompletely understood. Obesity-induced dysbiosis may disrupt eCBome homeostasis by reducing the availability of protective lipid mediators such as PEA and OEA, while promoting CB1 hyperactivation and increasing AEA levels. In turn, eCBome dysfunction and dysbiosis reinforce one another, contributing to metabolic alterations that further perpetuate eCBome–microbiota axis dysfunction.

**Fig. 2:**
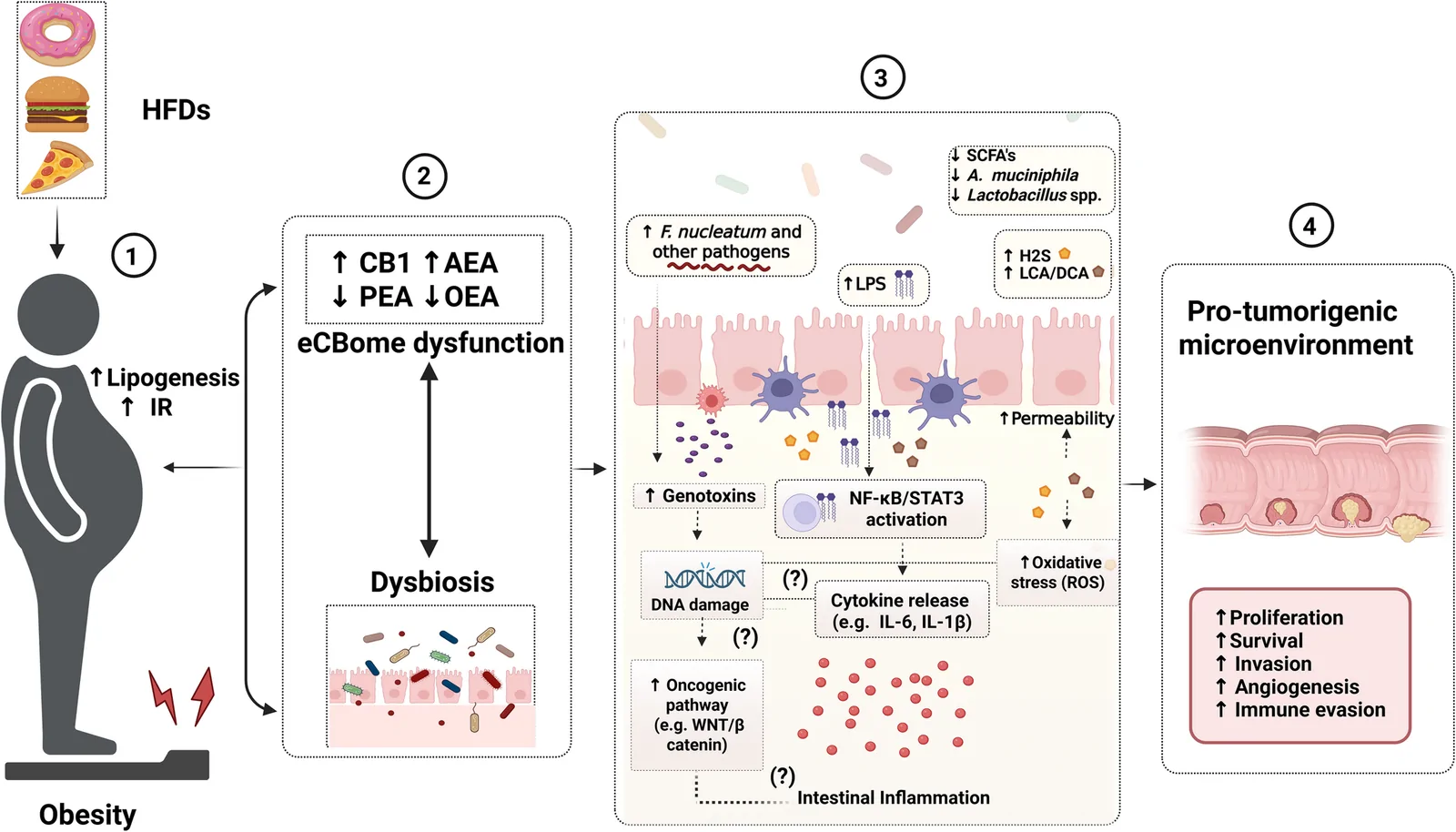
Potential link between eCBome–IM axis dysfunction in obesity and CRC development. HFDs promote obesity (1), which induces eCBome dysfunction and gut dysbiosis (2). These alterations are interconnected and may contribute to metabolic disturbances, including IR and increased lipogenesis, further reinforcing obesity and eCBome–microbiota axis dysfunction. As a result, SCFA-producing bacteria, such as *A. muciniphila*, decline, whereas potential pathogens (e.g., *F. nucleatum*) and harmful bacterial metabolites (e.g., LPS, H₂S, DCA, and LCA) increase. Together, these alterations impair intestinal barrier integrity, increase intestinal permeability, and promote oxidative stress (ROS), DNA damage, activation of inflammatory signaling pathways (NF-κB/STAT3), cytokine release, and oncogenic pathways (e.g., WNT/β-catenin), ultimately sustaining chronic intestinal inflammation (3). Collectively, these mechanisms may establish a pro-tumorigenic microenvironment characterized by enhanced proliferation, survival, invasion, angiogenesis, and immune evasion (4). The figure represents a hypothetical integrative model based on the current evidence linking obesity-associated eCBome–microbiota axis dysfunction to intestinal inflammation and colorectal carcinogenesis. Additional experimental studies are required to validate and further elucidate the proposed interactions. **HFDs**, high-fat diets; **IR**, insulin resistance; **SCFAs**, short-chain fatty acids; **AEA**, anandamide; **PEA**, palmitoylethanolamide; **OEA**, oleoylethanolamide; **LPS**, lipopolysaccharide; **H₂S**, hydrogen sulfide; **DCA**, deoxycholic acid; **LCA**, lithocholic acid; **NF-κB**, nuclear factor kappa B; **STAT3**, signal transducer and activator of transcription 3; **WNT/β-catenin**, WNT/β-catenin signaling pathway; **ROS**, reactive oxygen species. Created in https://BioRender.com

These alterations favor the expansion of potential pathogens, such as *F. nucleatum*, and increase the production of harmful bacterial metabolites (e.g., LPS, H₂S, DCA, and LCA), while reducing SCFA-producing bacteria, including *A. muciniphila.* Together, these changes impair intestinal barrier function and promote oxidative stress, DNA damage, activation of inflammatory signaling pathways (NF-κB/STAT3), and oncogenic pathways such as WNT/β-catenin. Collectively, these events sustain chronic intestinal inflammation and may establish a pro-tumorigenic microenvironment characterized by enhanced proliferation, survival, invasion, angiogenesis, and immune evasion (Fig. 2). This figure represents a hypothetical integrative model based on the current evidence, and the proposed interactions require further experimental validation.

## Changes in endocannabinoid signaling in CRC

Since obesity and microbiota-driven inflammatory changes may converge on endocannabinoid signaling, understanding how this system is altered during CRC progression is essential to define its potential role within the eCBome–microbiota–CRC axis. During colorectal carcinogenesis, CB1 expression progressively decreases in enterocytes, possibly due to hypermethylation of CpG islands in its promoter region [18]. This downregulation has been observed in both in vitro and in vivo CRC models [90–92], supporting a potential antitumor role for CB1 in specific contexts.

Tutino et al. [92] reported that CB1 expression is reduced in tumor tissue and adjacent mucosa of patients with metastatic CRC, accompanied by inhibition of MAPK-p38/ERK1/2 (pathways involved in apoptosis and regulation of cell proliferation), activation of PI3K/Akt, and lower levels of the pro-apoptotic protein Bax, suggesting that reduced CB1 contributes to CRC progression. Similarly, deletion of CB1 in *Apc*^Min/+^ mice promoted intestinal polyp formation, underscoring its crucial role in maintaining intestinal homeostasis and protecting against colorectal tumorigenesis [91].

CB2 has been shown to exert a protective effect in CRC. In various murine models of CRC (spontaneous, AOM/SDS-induced, and *Apc*^Min/+^), CB2^−/−^ mice displayed a more pro-tumorigenic immune environment, including higher incidence of precancerous lesions and tumors, elevated IL-6 levels, increased marrow-derived myeloid-derived suppressor cells (MDSCs) infiltration, and reduced CD8⁺ T cells. These changes contributed to immunosuppression, angiogenesis, tumor invasion, and metastasis. Similarly, in humans, *CNR2* gene mutations were significantly associated with colon cancer incidence [93]. Building on this evidence, in vitro studies demonstrated that CB2 activation induces apoptosis in CRC cells through de novo ceramide synthesis [94]. Furthermore, the selective CB2 agonist JWH-133 reduced viability and promoted apoptosis in HT-29 cells [95], suggesting a protective role for CB2 in colorectal cancer.

Although cannabinoid receptors can have protective roles in CRC, their effects are context-dependent, influenced by tumor microenvironment, disease stage, receptor expression, and agonist type and concentration [18]. For instance, elevated CB1 expression was associated with poorly differentiated tumors and worse prognosis in patients with stage II microsatellite instability-high (MSI-H) [96], possibly due to the biphasic nature of CB1 signaling, where low ligand levels activate pro-apoptotic pathways (ERK) and high levels promote cell survival via AKT [96, 97]. Similarly, CB2 activation with submicromolar concentrations of selective agonists (JWH-133, HU-308) stimulated HCT116 cell proliferation and tumor growth in vivo via AKT activation and E-cadherin degradation [98]. These findings highlight the complex, context-specific roles of cannabinoid receptors in CRC and suggest their expression could serve as a biomarker of tumor progression [18].

Unlike cannabinoid receptors, GPR55 acts as a protumor modulator in CRC, particularly in colitis-associated AOM/SDS models. Its activation alters MDSCs and T lymphocytes and enhances proinflammatory molecules such as COX-2 and STAT3, promoting tumor initiation and progression. Consistent with this, GPR55^−/−^ mice show reduced tumor burden, and levels of its endogenous ligand L-α-lysophosphatidylinositol (LPI) are elevated in CRC patients, highlighting GPR55’s key role in inflammation-driven tumorigenesis [96, 98].

Studies have shown significant variations in the levels of eCBs at different stages of colorectal tumorigenesis [93, 99–101]. For example, Ligresti et al. [90] reported 2–3 fold higher AEA and 2-AG levels in colorectal adenomas and carcinomas compared to normal mucosa, with greater increases in adenomas. In AOM-treated mice, 2-AG levels increased and were correlated with aberrant crypt foci and preneoplastic colonic lesions [102]. In human colorectal tumors, AEA and its metabolite AA were significantly elevated, particularly in patients with lymphatic metastasis [99]. However, elevated eCB levels should be interpreted cautiously, as they may reflect compensatory, inflammatory, or tumor-associated changes depending on the stage and biological context of CRC, rather than a direct contribution to tumor progression.

In line with this context-dependent behavior, studies have suggested both tumor-limiting and pro-apoptotic effects of eCB signaling in CRC models. For example, elevated plasma 2-AG levels have been observed in CRC patients. However, whether this increase represents a compensatory antitumor response or simply reflects inflammatory or tumor-associated changes remains uncertain [103]. In contrast, FAAH inhibition in AOM-treated animals increased AEA and 2-AG levels, reduced ACF formation, and partially restored cleaved caspase-3 expression, indicating enhanced apoptosis [102]. Likewise, increasing concentrations of AEA inhibited proliferation in human CRC cell lines through CB1 activation and were associated with decreased polyamine levels [100].

One of the mechanisms by which eCBs exert anti-inflammatory and antitumor effects in the intestine is through the suppression of pro-inflammatory mediators, including TNF-α, IFN-γ, IL-1β, and IL-6 [18]. Their antitumor activity is further enhanced via metabolism by the COX-2 enzyme, which generates prostaglandin ethanolamides (PG-EAs). These metabolites display pro-apoptotic effects in colorectal cancer cell lines with high COX-2 expression, such as HT-29 and HCA7/C29, while simultaneously limiting arachidonic acid availability for PGE2 synthesis, a prostaglandin involved in tumor promotion [101].

Finally, several enzymes involved in eCB synthesis and degradation exhibit altered expression in CRC [100, 103]. FAAH and NAPE-PLD mRNA levels are increased in the tumor tissues of patients with CRC, indicating altered expression of enzymes involved in AEA metabolism. Functionally, FAAH inhibition in animal models reduced precancerous lesions, possibly through increased eCB levels [74, 102]. Moreover, FAAH overexpression was associated with increased levels of AA, which may favor inflammatory pathways implicated in CRC development [99].

## Conclusion

The eCBome–microbiota axis is a bidirectional regulatory network integrating host and microbial signals, with key roles in maintaining intestinal homeostasis and regulating metabolic, immune, and barrier functions relevant to CRC development. In this context, obesity-related dysfunction of this axis may represent an underexplored mechanistic pathway that could contribute to a pro-tumorigenic intestinal environment and help contextualize why obesity is recognized as an established risk factor for CRC, although most evidence to date remains preclinical or associative, with no established causal relationships.

Overall, this framework provides a novel conceptual perspective for CRC prevention and therapy, shifting the focus from individual factors such as microbiota composition or inflammatory signaling alone toward their integrated regulation through the eCBome–microbiota interface. From a translational standpoint, this review supports the hypothesis that interventions targeting the eCBome–microbiota axis, including dietary modulation, physical activity, probiotics, and pharmacological approaches, may represent promising strategies to restore its balance and potentially confer protection against CRC, particularly in obesity-driven CRC, although this protective effect remains to be experimentally tested.

## Future perspectives

The recognition that the eCBome and gut microbiota can mutually influence each other opens new perspectives for therapeutic strategies across different conditions, given the relevance of this axis in physiological regulation. In the context of obesity-induced CRC, this review compiles preclinical evidence supporting the hypothesis that the eCBome–microbiota axis represents a promising therapeutic pathway.

Future studies should further clarify how eCBome components and microbial communities interact across the stages and molecular subtypes of CRC, including their effects on the tumor microenvironment and immune regulation. In particular, studies focusing on the interaction between the microbiota and its metabolites and CB1 and CB2 signaling across different CRC subtypes and stages may clarify their contributions to tumor suppression or progression. Furthermore, future research should also explore whether components of the eCBome–microbiota axis can serve as biomarkers for CRC risk stratification, prognosis, or treatment response, particularly across different CRC subtypes and stages.

From a translational perspective, specific probiotics, such as *A. muciniphila*, *L. acidophilus*, and *L. johnsonii*, represent promising candidates for therapeutic strategies due their ability to modulate eCBome components, restore barrier function, and influence CRC-related signaling pathways. However, emerging evidence suggests that *A. muciniphila* may exert context-dependent effects influenced by host microbiota composition, disease stage, and mode of administration [104], underscoring the need for caution in its therapeutic application. In parallel, combined strategies targeting both the eCBome and gut microbiota, including dietary patterns such as the Mediterranean diet, may enhance the production of SCFAs and eCB-related mediators such as 2-MAGs and PEA.

Finally, investigating epigenetic regulation and early-life metabolic programming as modulators of the eCBome–microbiota axis represents a novel and emerging area of research. In this context, early-life exposures may induce long-lasting changes in host–microbial interactions, shaping eCBome signaling, immune system maturation, and intestinal homeostasis, with potential consequences for CRC risk. This research area is particularly relevant as it may reveal new anticancer pathways and early targets for prevention, especially given the increasing incidence of CRC in individuals under 50 and the need to better understand disease onset and strengthen primary prevention strategies for early-onset CRC.

## Data Availability

No datasets were generated or analysed during the current study.
